# Stem Tones Pre-activate Suffixes in the Brain

**DOI:** 10.1007/s10936-016-9434-2

**Published:** 2016-05-30

**Authors:** Pelle Söderström, Merle Horne, Mikael Roll

**Affiliations:** Centre for Languages and Literature, Lund University, Box 201, 221 00 Lund, Sweden

**Keywords:** Pre-activation, Prosody, ERP, Morphology, Speech processing

## Abstract

Results from the present event-related potentials (ERP) study show that tones on Swedish word stems can rapidly pre-activate upcoming suffixes, even when the word stem does not carry any lexical meaning. Results also show that listeners are able to rapidly restore suffixes which are replaced with a cough. Accuracy in restoring suffixes correlated positively with the amplitude of an anterior negative ERP elicited by stem tones. This effect is proposed to reflect suffix pre-activation. Suffixes that were cued by an incorrect tone elicited a left-anterior negativity and a P600, suggesting that the correct processing of the suffix is crucially tied to the activation of the preceding validly associated tone.

## Introduction

### Tonal Cues and Pre-activation of Grammatical Structures

To facilitate rapid processing of sensory input, the brain constantly makes predictions about future events (Bar 2007). In language processing, intonation has been seen to provide cues for upcoming syntactic and lexical structures (Kjelgaard and Speer 1999; Steinhauer et al. 1999; Roll et al. 2009, 2011; Hawthorne and Gerken 2014; Hirose and Mazuka 2015). Still, it is not known how the brain handles tonal cues for upcoming grammatical structures within single words. For Swedish, it has been suggested that tones on word stems (“word accents”) are used as cues to pre-activate upcoming suffixes (Roll et al. 2015). Suffixes that are invalidly cued by an incorrect tone have been seen to increase response times and lead to a P600 reprocessing effect in event-related potential (ERP) studies (Roll et al. 2010; Söderström et al. 2012; Roll et al. 2013; Roll 2015; Roll et al. 2015). However, it is unclear whether this tone-morphology interaction is based on a direct association between tones and suffixes in the brain, independent of their association with lexical word stems, or whether the tone-suffix association is dependent on and tied to whole word forms. Furthermore, we do not know whether this tone-suffix connection actually involves pre-activation of the suffix. The present ERP study was the first to use pseudowords rather than existing words to test the hypothesis that there is a pure tone-suffix association which exists even in the absence of a lexical word stem. The nature and strength of this association was investigated by comparing processing of suffixes as well as conditions where suffixes were masked by a light cough attached to stems. If test participants can recover the meaning of suffixes based on the tone alone, this would suggest that the tone-suffix association is independent of word stem content.

### Word Tones in Swedish

Central Swedish has two tones: accent 1, a low tone on the stressed vowel of the word stem, and accent 2, a high tone on the stressed stem vowel. The tones are morphologically conditioned, so that certain suffixes induce accent 1 onto the word stem while others induce accent 2. For example, a noun like $b\mathring{a}t$ (‘boat’) with the singular suffix *-en* is associated with accent 1 on the stem ($b\mathring{a}t_{1}\hbox {-}en$ ‘boat-the’) while plural nouns ending with the suffix *-ar* take accent 2 ($b\mathring{a}t_{2}\hbox {-}ar$ ‘boat-s’). For verbs, the present tense suffix *-er *induces accent 1 onto the word stem ($t\ddot{a}nk_{1}\hbox {-}er$ ‘think.PRES’) while past tense *-te *induces accent 2 ($t\ddot{a}nk_{2}\hbox {-}te$ ‘think.PAST’). Accent 1 seems to be a stronger suffix predictor than accent 2, since subjects have been seen to respond faster to validly cued accent 1 suffixes than accent 2 suffixes (Söderström et al. 2012). This follows from the fact that accent 2 is also associated with compound words, meaning that accent 2 on a stem such as $b\mathring{a}t_{2}$ (‘boat’) could signal either a compound such as $b\mathring{a}t_{2}\hbox {-}hus$ (‘boat-house’) or a suffixed word (Riad 2012). Accent 1 thus cues a smaller set of possible forms: hearing an accent 1 on the stem $b\mathring{a}t_{1}$ indicates that a member of a well-defined set of suffixes will follow, e.g. the singular suffix *-en*.

### Suffix Activation by Tones

In ERP studies, accent 1 stems have invariably and independently of task elicited a left to mid anterior negativity as compared to accent 2 stems (Roll et al. 2010, 2013, 2015; Roll 2015). In previous studies, this effect has been viewed as a positively charged effect for accent 2. Data from Roll et al. (2015), however, has led to a re-evaluation of this view. There it was found that accent 1 stems gave rise to increased neural activity, as evidenced by a greater BOLD (blood-oxygen-level dependent) effect as well as greater gRMS (global root mean squares) amplitudes for accent 1 compared to accent 2.^1^ It is not clear, however, just what this effect reflects. It is not due to an acoustic difference between word accent tones, since it has also been found for South Swedish, in which the word accents are the mirror image of those in Central Swedish: accent 1 can be analysed as a high tone while accent 2 is a low tone (Roll 2015). Furthermore, Roll et al. (2013) found no negativity for de-lexicalised accent 1 stimuli, but rather an increased N1 component for accent 2 stimuli, due to their greater perceptual salience (high tone). The negativity has also been found for both nouns and verbs, indicating that the word accents are not tied to specific grammatical properties such as number or tense.

One suggested explanation for the negativity effect is that it indexes automatic suffix pre-activation by the tone and that it is modulated by the ease with which upcoming linguistic information can be activated. In the fMRI investigation reported on in Roll et al. (2015), activation for accent 1 was found in BA47 in the inferior frontal gyrus (IFG), an area known to be involved in morphological processing. This effect—as well as an effect in primary and secondary auditory cortex—correlated with a gRMS peak at 136 ms after tone onset. This could suggest a mechanism by which the tone activation in auditory cortex initiates suffix processing modulated by the IFG even before the suffix has been heard. This has led us to assume that morphological processing is initiated ahead of time and that the suffix could be pre-activated. If the pre-activating mechanism operates solely on the tone-suffix association rather than on whole word forms, a similar effect should be expected for words with pseudo-stems. Furthermore, in line with previous studies, suffix pre-activation would be expected to be stronger for accent 1 as compared to accent 2.

To test whether tones pre-activate suffixes and whether listeners are able to recover the grammatical ending on the basis of tone alone, the present study used both existing suffixes and endings where coughs replaced suffixes. The task was to determine whether the pseudoword (pseudo-stem plus valid or invalid suffix, or cough) was singular or plural. If the accent 1 negativity reflects a greater degree of suffix pre-activation, one would expect to find higher accuracy rates when listeners guess the meaning of cough-endings following accent 1 as compared with accent 2. A positive correlation between the ERP negativity amplitude and response accuracy would also be expected.

### Reanalysis of Invalid Suffixes

In line with earlier studies, we expect invalidly cued suffixes to elicit a P600 effect reflecting reanalysis of the incorrect form. In particular, combinations of accent 1 stems and invalidly cued suffixes have been seen to give rise to the greatest P600 amplitudes. Furthermore, closer inspection of the ERPs in Roll et al. (2013) suggests an increased negative deflection (in the 200–400 ms time window after suffix onset) which preceded the P600 for suffixes invalidly cued by the wrong word accent. It was therefore hypothesised that invalidly cued suffixes in the present study would elicit a left-anterior negativity (LAN), reflecting increased morphological processing (Penke et al. 1997; Weyerts et al. 1997).

#### Effects of Cough-Ending

Since cough-endings constitute novel, unexpected events, they could be thought to elicit a surprisal effect, seen as an increase in the P3a component (Polich 2007) as compared to suffixes. The amplitude of the P3a would be expected to vary along with response accuracy, so that participants who can be argued to have pre-activated the suffix more strongly (as indicated by response accuracy) should also show an increased surprise response (P3a) when a cough instead of a suffix is perceived.

## Materials and Methods

### Stimuli

**Fig. 1:**
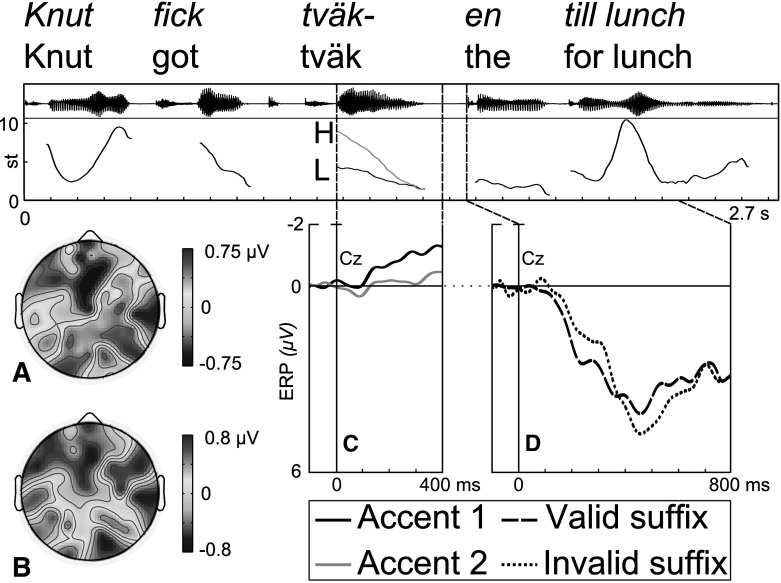
*Top* example of the test sentences used in the present study with sound waveform and fundamental frequency (F0) curve, (st $=$ semitones, H $=$ “high tone”, L $=$ “low tone”). **a** Subtraction plot showing the negativity elicited by Accent 1 stems at 200–400 ms following tone onset. **b** Subtraction plot showing the left-anterior negativity (LAN) found at 200–400 ms after onset of invalidly cued suffixes. **c** ERP waveforms for all Accent 1 (*black line*) and Accent 2 (*grey line*) stems, where the negativity for Accent 1 is visible. **d** ERP waveforms to valid (*dashed line*) and invalid (*dotted line*) suffixes, showing a negativity at 200–400 ms (LAN) followed by a positivity at 400–700 ms (P600)

The stimuli were phonotactically possible pseudowords, with a CCV:C structure (e.g. $tv\ddot{a}k$ [$\hbox {tf}\upvarepsilon {:}\hbox {k}$]), associated with accent 1 and 2. All pseudo-stems ended with an unvoiced plosive to facilitate splicing. In an offline pre-test using the 40 pseudowords, along with 40 pseudoword fillers and 40 existing words with the same syllable structure, 38 Swedish speakers—none of whom participated in the main study—assessed whether the stimuli were real Swedish words, using a 0–10 point scale where 0 was “not real” and 10 was “real”. Existing words, $M = 8.5$, received significantly higher test scores than pseudowords, $M = 1.6, t(2193.0) = 94.6$, $p < 0.001$. The pseudowords were embedded in sentences recorded in an anechoic chamber by a male Central Swedish speaker. An example sentence is *Knut fick tväken/tväkar till jul* ‘Knut got the tväk/tväks for Christmas’ (Fig. 1).

The pseudowords were split into a stem and suffix using Praat (Boersma 2001). Accent 1 and 2 pseudo-stems measured from word onset to vowel offset had very similar overall duration, $M = 472/467\,\hbox {ms}$, and vowel duration, $M = 266/249\,\hbox {ms}$. Fundamental frequency (F0) fall during the vowel was greater for accent 2, $M = 6.7$ semitones, than for accent 1, $M = 2.4$ semitones. The post-vocalic consonant (plosive)—and thus place of articulation—was the same across conditions (e.g. *tvä-ken*/*tvä-kar*). The mean duration of singular (accent 1-associated) and plural (accent 2-associated) suffixes was 300 and 284 ms, respectively. All stimuli were normalised for intensity. After normalisation, there was a slight difference in intensity between accent 1 ($M = 70.4\,\hbox {dB}$) and accent 2 ($M = 71.8\,\hbox {dB}$) pseudo-stems, due to the natural relation between pitch height and intensity (Stevens 1935). Valid, invalid and cough-ending pseudoword forms were created by cross-splicing (see Table 1 for details). Conditions with cough-endings were created by splicing a coughing sound recorded by the same speaker onto pseudo-stems (e.g. *tvä-cough*). The cough matched the mean duration and intensity of the singular and plural suffixes in the study. There were 40 pseudowords per condition and 6 conditions, for a total of 240 stimuli. Suffix onset in this study was onset of the plosive (-*ken* in *tväken*) or onset of the cough. This was the disambiguation point at which participants could judge whether the pseudoword was singular or plural. The sentences were identical up until and after the pseudoword in all conditions. Thus, the tone was the first reliable cue with which to predict the suffix.

**Table 1:** The six conditions used in the present experiment, resulting from combinations of stem tones with suffixes and cough-endings

	Suffix 1	Suffix 2	Cough
Accent 1	Acc1Valid	Acc1Invalid	Acc1Cough
Accent 2	Acc2Invalid	Acc2Valid	Acc2Cough

### Experimental Setup

#### Audiometric Testing

A Békésy audiometric test was administered to all participants ($N = 17$) to estimate their pure-tone hearing thresholds for frequencies 250–8000 Hz, using a GN Otometrics Astera audiometer and Sennheiser HDA 200 earphones (Brännström and Grenner 2008, tone $\hbox {duration} = 250\,\hbox {ms}$, intensity rate $\hbox {change/second} = 2\,\hbox {dB}$). The setup was calibrated in accordance with IEC 60318-2 and ISO 389-8 (IEC 60318-1 1998a; IEC 60318-2 1998b; ISO 2004). Three participants were assessed to be beneath the threshold of normal hearing (defined as pure-tone hearing thresholds $\le$20 dB Hearing Level (ISO 2004) for frequencies 250–8000 Hz) and were excluded from the study, leaving 14 participants (mean age: 24.7 years ($SD = 4.7$ years), 7 women) for further analysis. All participants were right-handed (Oldfield 1971), Central Swedish speakers.

#### Sound Presentation and Calibration

The stimuli were presented at 70 dB using E-Prime on a PC with an internal soundcard (16 bits/44.1 kHz) connected to the audiometer and Eartone 3A insert earphones. The complete equipment setup was calibrated in accordance with IEC 60318-4 and ISO 398-2 (ISO 1994; IEC 2008). A 1000 Hz tone with average RMS equal to that of the speech signals was used for stimulus calibration.

#### Electroencephalography

The EEG was recorded using a 129-channel HydroCel Geodesic Sensor Net from Electrical Geodesics Incorporated using the same settings and electrode regions as in Roll et al. (2013). Epochs were extracted beginning from critical word F0-onset (500 ms duration) to compare the word accents, and from suffix onset (800 ms duration) to analyse the Suffix and Cough conditions. A 100 ms prestimulus time window was used for baseline correction. Epochs exceeding ±100 $\upmu \hbox {V}$ after compensation for eye artifacts using ICA (independent component analysis (Jung et al. 2000)) were rejected, mean 14 % ($SD = 8\,\%$) for word F0-onset and 24 % ($SD = 15\,\%$) for suffix onset. Greenhouse–Geisser correction was used when applicable. A 15 Hz presentation filter was used for visualisation purposes.

#### Procedure

Participants were seated in front of a computer screen and looked at a fixation cross while listening to sentences. They pressed one of two buttons as quickly as possible to indicate whether the pseudoword was singular or plural (including pseudowords with cough-endings). The order of the buttons was counter-balanced across experimental blocks. There were four blocks in total, with 60 stimuli in each. Stimulus presentation within blocks was pseudo-randomised and optimised using OptSeq2 (Dale 1999). Stimulus onset asynchrony was jittered between 2 and 14 s. Different OptSeq sequences were used for each block. Before the experiment, participants underwent a short training period. The training block included both Suffix and Cough conditions. Participants were told that the critical words in the task had no meaning, but that they would be able to guess their number (since the endings were existing singular (*-en*) and plural (*-ar*) suffixes). They were also told that some suffixes would be replaced with coughs.

## Results

### Behavioural Data

Factors used for analysing response times to suffixed conditions ($\hbox {Acc1Valid} = 633\,\hbox {ms}$ ($SD = 66$), $\hbox {Acc2Valid} = 655\,\hbox {ms}$ ($SD=60$), $\hbox {Acc2Invalid} = 693\,\hbox {ms}$ ($SD= 62$), $\hbox {Acc1Invalid} = 658\,\hbox {ms}$ ($SD=59$)) were validity of the tone-suffix combination (valid, invalid) and suffix (singular, plural). There was a validity main effect ($F(1,13) = 6.203$, $p = 0.027$) and a validity $\times$ suffix interaction ($F(1,13) = 19.006$, $p = 0.001$). The singular suffix *-en* led to increased response times when invalidly cued by accent 2 stems as compared to when it was correctly cued by accent 1 ($F(1,13) = 11.635$, $p = 0.005$). Response accuracy for the four suffixed conditions ($\hbox {Acc1Valid} = 97.3\,\%$ ($SD= 1.4$), $\hbox {Acc1Invalid}=93.1\,\%$ ($SD = 3.4$), $\hbox {Acc2Invalid} = 89.9\,\%$ ($SD = 3.8$), $\hbox {Acc2Valid} = 98.6\,\%$ ($SD= 0.8$)) displayed a main effect of validity ($F(1,13) = 7.280$, $p = 0.018$). Invalidly cued suffixes led to lower response accuracy compared to validly cued suffixes.

Mean response times for the Cough condition were 792 ms ($SD =71$) for accent 1 and 798 ms ($SD = 64$) for accent 2. Response accuracy was well above chance for both accents but accuracy was significantly higher for accent 1 (87.8 %, $SD = 2.8$) compared to accent 2 (72.0 %, $SD = 4.8$) ($F(1,13) = 11.235$, $p < 0.01$).

### ERP Data

#### F0-Onset (200–400 ms After F0-Onset)

The additional factors used for the ERP analyses were antpost (anterior, central, posterior regions) and laterality (left, mid, right regions) and the dependent variable was mean amplitude. Since stems for all conditions were identical up until onset of the plosive, ERPs for all stems were averaged and used for the stem ERP analyses. ERPs in response to accent 1 and 2 stems started to diverge around 200 ms following F0-onset of the pseudoword (Fig. 1).^2^ Accent 1 elicited a negativity compared to accent 2. A tone $\times$ antpost $\times$ laterality interaction was found ($F(4,52) = 4.015$, $p= 0.021$). There were tone $\times$ antpost interactions at left ($F(2,26) = 7.614$, $p = 0.003$) and mid ($F(2,26) = 6.808$, $p = 0.017$) electrodes. Accent 1 yielded significantly more negativity than accent 2 over left anterior ($F(1,13) = 5.843$, $p= 0.031$) and mid anterior sites ($F(1,13) = 6.619$, $p = 0.023$).

#### Suffix Onset (200–400 ms After Onset)

At 200–400 ms after suffix onset, an analysis of validly and invalidly cued suffixes was performed to investigate the possible LAN effect. A validity $\times$ antpost $\times$ laterality interaction was found ($F(4,52) = 6.773$, $p= 0.002$). There was a validity $\times$ laterality interaction ($F(2,26) = 9.064$, $p= 0.004$) at anterior sites. Invalidly cued suffixes produced significantly more negativity than validly cued suffixes over left anterior ($F(1,13) = 6.778$, $p = 0.022$) and mid anterior ($F(1,13) = 7.290$, $p= 0.018$) sites, suggestive of a left-anterior negativity for invalidly cued suffixes.

**Fig. 2:**
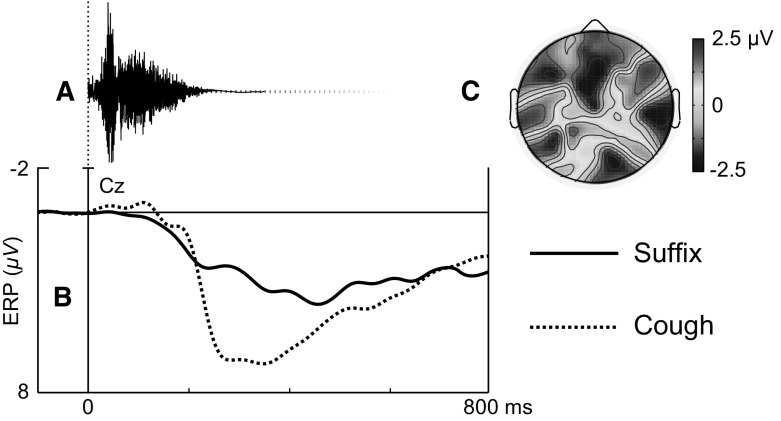
**a** Cough-ending sound waveform. **b** ERP waveforms to matching suffixes (*black line*) compared to cough-endings (*dotted line*). **c** Subtraction plot showing the topographic distribution of the P3a for cough-endings compared to suffixes at 200–400 ms

To analyse the ERP response to cough stimuli, an analysis using the factor ending (validly cued suffix, cough) was performed in the 200–400 ms window. An ending $\times$ antpost $\times$ laterality interaction was found ($F(4,52) = 21.513$, $p < 0.001$). There were ending $\times$ laterality interactions at anterior ($F(2,26) = 15.749$, $p < 0.001$) and central ($F(2,26) = 27.819$, $p < 0.001$) sites. ERPs in response to coughs were significantly more positive as compared to suffixes over left anterior ($F(1,13) = 28.723$, $p < 0.001$), mid anterior ($F(1,13) = 21.458$, $p < 0.001$) and mid central ($F(1,13) = 70.860$, $p < 0.001$) sites (see Fig. 2). This effect is most likely a P3a, obtained due to the surprising and novel nature of the coughs (Polich 2007). No significant differences were found in the P3a amplitude when accent 1 and 2 words were compared.

#### Suffix Onset (400–700 ms After Onset)

A window of 400–700 ms after suffix onset was extracted to analyse late positivities in response to invalidly cued suffixes. A validity $\times$ antpost $\times$ laterality interaction was found ($F(4,52) = 3.204$, $p = 0.047$). A validity $\times$ laterality interaction was found at posterior sites ($F(2,26) = 7.071$, $p = 0.014$). There were effects of validity at left posterior ($F(1,13) = 11.050$, $p= 0.005$) and mid posterior ($F(1,13) = 8.885$, $p = 0.011$) sites, suggesting a P600 effect for invalidly cued suffixes in general. There were no validity $\times$ tone interactions and consequently no significant differences in the P600 between Acc1Invalid and Acc2Invalid.

#### ERP Amplitude Correlations in the Cough Condition

To further investigate the accent 1 negativity, a correlation analysis was conducted using mean ERP amplitudes per subject in the 200–400 ms F0-onset window (left and mid anterior sites) and mean response accuracy per subject in the Cough condition. One subject was excluded due to excessively low response accuracy. Increased left-anterior negativity for accent 1 stems correlated with higher response accuracy for accent 1 followed by cough-endings (Pearson’s $r = -0.448$, $p = 0.022$).

Furthermore, correlation analyses were carried out to investigate subject variability in the amplitude of the P3a component over mid central sites found in response to cough stimuli. A positive correlation (Pearson’s $r= 0.650$, $p= 0.012$) was found between response accuracy and P3a amplitude for Acc1Cough, meaning that participants with higher response accuracy exhibited larger P3a amplitudes. No correlations were found for accent 2 stems.

## Discussion

The results indicate that suffixes can be pre-activated by stem tones to which they have strong neural connections. Furthermore, since the results were found for pseudowords, this pre-activation seems to operate on the tone-suffix association independently of lexical content. In addition to finding ERP components (LAN/P600) elicited by suffixes which were invalid with respect to the preceding tone, the present study also found evidence in support of a possible suffix pre-activation mechanism. We found a left frontal negativity for accent 1 which correlated with accuracy in guessing suffixes replaced by coughs, supporting the view that accent 1 is a stronger predictor of its associated suffixes than accent 2. Both accent 1 and accent 2 do seem to be used for suffix pre-activation, as evidenced by the relatively high accuracy rates for both stem tones when participants guessed which suffix the cough-ending replaced. However, accent 1 stems cue a much smaller number of possible continuations, since accent 2 stems can also be associated with compounds. Therefore, accent 1 endings can be more easily accessed as compared to continuations following accent 2 stems.

Two ERP effects in two different time windows were found after suffix onset. Pseudowords with invalid tone-suffix combinations elicited a P600-like effect in the 400–700 ms time window, reflecting reanalysis triggered by the mismatch. In the 200–400 ms suffix onset time window, a left to mid anterior negativity was found for invalid suffixes. This was interpreted as a left-anterior negativity (LAN), an effect related to morphosyntactic processing and structure building which possibly reflects the activation of an unprimed cortical memory trace of a suffix (Pulvermüller and Shtyrov 2003).

ERP subtraction plots reveal similarities between the scalp distribution and time course of the accent 1 negativity and the LAN (see Fig. 1), suggesting that these effects might perhaps both reflect morphological processing: due to the high predictive value of accent 1, the tone-suffix memory trace can be more strongly pre-activated upon hearing an accent 1 stem, and this is reflected in an increased left to mid anterior negativity. These similarities could possibly be used to argue that the accent 1 negativity is an index of pre-activation triggered by the tone, while the LAN–which was temporally and spatially similar to the preceding stem negativity–reflects increased morphological processing triggered by an unexpected suffix. Furthermore, there was a strong correlation between the Acc1Cough response accuracy and P3a amplitude. Participants who restored suffixes more accurately can also be argued to have pre-activated these suffixes more strongly, leading to an increased surprise effect when the stem is followed by a cough. Together with the finding that the amplitude of the accent 1 negativity correlates with response accuracy to cough-endings, these results lead us to suggest that accent 1 pre-activates suffixes more strongly than accent 2.

## Conclusions

The present study provides evidence that the brain can use word tones to activate morphological information ahead of time. Furthermore, a LAN was found for invalid tone-suffix combinations, suggesting that suffixes are dependent on the stem tone in order to be optimally activated.

We propose that listeners can use neural tone-suffix connections to rapidly pre-activate upcoming suffixes. We have shown that this connection is independent of the lexical status of the stem. The association between phonological and morphological information has even been seen to be strong enough to allow listeners to use tones to fully recover suffixes when they are replaced with coughs.

## Footnotes

1 Although gRMS peaks are likely to stem from increased neural activity, they could also be due to other factors, e.g. increased neural synchronisation (Skrandies 1990).

2 One reason for a slightly later effect compared to e.g. Roll et al. (2015) was that the present study used words in which vowel onset occurred later.
